# Exercise-based cardiac rehabilitation for patients with angina pectoris - a CaReMATCH individual participant data meta-analysis

**DOI:** 10.1016/j.ajpc.2026.101535

**Published:** 2026-03-10

**Authors:** Iris A. De Koning, Joyce.M. Heutinck, Benjamin J.R. Buckley, Grace O. Dibben, Gianluca Campo, Arto J. Hautala, Johan A. Snoek, Ralph Maddison, Núria Santaularia, Scott A. Lear, Julie Houle, Gregory Y.H. Lip, Robert Jan M. Van Geuns, Tom Vromen, Hareld M.C. Kemps, Rod S. Taylor, Niels A. Stens, Dick H.J. Thijssen, CaReMATCH collaborators, Alun D. Hughes, Ajay S. Vamadevan, Ambalam M Chandrasekaran, Ambuj Roy, Andrea Raisi, Anil R Jain, Arnoud W.J. van’t Hof, Bhaskara Rao, Bishav Mohan, Davinder S Chadha, Dimple Kondal, Divya Soni, Dorairaj Prabhakaran, Ed P. de Kluiver, Elisabetta Tonet, Gianni Mazzoni, Giovanni Grazzi, Ian Roberts, Jabir Abdullakutty, Kalpana Singh, Kaushik Chattopadhyay, Kavita Singh, Kolli S Reddy, Kushal Madan, Manjunath C Nanjappa, Nagamalesh U Madappa, Nagraj Desai, Narsimhan Calambur, Natrajan KU, Nikhil Tandon, Nishi Chathurvedi, Paul Poirier, Poppy Malinson, Prabhavathi Bhat, Pradeep A Praveen, Prakash C Negi, Prasad MR, Raghava Sarma, Raji Devarajan, Ravindra K Tongia, Rita Pavasini, Sadananda S Kanchanahalli, Sanjay Kinra, Satish Patil, Shah Ebrahim, Shankar Patil, Sharad Chandra, Srikumar Swaminathan, Srinivas Mallya, Stefano Volpato, Stuart Pocock, Subhash C Manchanda, Subramaniam Natarajan, Sudhir R Naik, Sunil Kumar

**Affiliations:** a1 MRC Unit for Lifelong Health and Ageing at University College London, London, United Kingdom; a2 Centre for Chronic Disease Control, New Delhi, India; a3 Public Health Foundation of India, New Delhi, India; a4 Centre for Chronic Disease Control, Public Health Foundation India, New Delhi, India; a5 All India Institute of Medical Sciences, New Delhi, India; a6 Center for Exercise Science and Sport, University of Ferrara, Ferrara, Italy; a7 SAL Hospital and Medical Institute, Ahmedabad, India; a8 Department of Cardiology, Maastricht University Medical Center and Cardiovascular Research Institute Maastricht (CARIM), Maastricht, the Netherlands; a9 CARE Hospital Heart institute, Vishakapatnam, India; a10 Dayanand Medical College, Ludhiana, India; a11 Command Hospital, Bengaluru, India; a12 Department of Non-communicable Disease Epidemiology, London School of Hygiene and Tropical Medicine, London, UK; a13 None; a14 Cardiology Unit, Azienda Ospedaliera Universitaria di Ferrara, Ferrara, Italy; a15 London School of Hygiene and Tropical Medicine, London, United Kingdom; a16 Lisie Hospital, Kochi, India; a17 University of Nottingham, Nottingham, United Kingdom; a18 Sir Ganga Ram Hospital, New Delhi, India; a19 Sri Jayadeva Institute of Cardiovascular Sciences and Research, Bengaluru, India; a20 M S Ramaiah Medical College and Hospital, Bengaluru, India; a21 JSS Medical College, Mysuru, India; a22 CARE Hospital, Hyderabad, India; a23 Amrita Institute of Medical Sciences, Kochi, India; a24 Imperial College London, London, United Kingdom; a25 Faculty of Pharmacy, Université Laval; a26 Institut universitaire de cardiologie et de pneumologie de Québec; a27 Indira Gandhi Medical College, Shimla, India; a28 KLE Academy of Higher Education & Research/ College/university, Belgaum, India; a29 Lalitha Super Specialities Hospital, Kothapeta, India; a30 Escorts Fortis Hospital, Jaipur, India; a31 Sri Jayadeva Institute of Cardiovascular Sciences and Research, Mysuru, India; a32 SRI BM.Patil medical college, Vijayapura, India; a33 King George’s Medical University, Lucknow, India; a34 Frontier Lifeline Hospital, Chennai, India; a35 Department of Medical Science, University of Ferrara, Ferrara, Italy; a36 G.Kuppuswamy Naidu Memorial Hospital, Coimbatore, India; a37 Apollo Hospital, Jubilee Hills, Hyderabad, India; a38 JSS Hospital, Mysuru, India; a Department of Medical BioSciences, Radboud University Medical Center, Nijmegen, the Netherlands; b Department of Industrial Design, Eindhoven University of Technology, Eindhoven, the Netherlands; c Liverpool Centre for Cardiovascular Science, University of Liverpool, Liverpool John Moores University, and Liverpool Heart & Chest Hospital, Liverpool, United Kingdom; d Research Institute for Sport and Exercise Sciences, Liverpool John Moores University, Liverpool, United Kingdom; e Primary Care and General Practice, University of Glasgow, Glasgow, United Kingdom; f Cardiology Unit, Azienda Ospedaliero Universitaria di Ferrara, Ferrara, Italy; g Faculty of Sport and Health Sciences, University of Jyvaskyla, Jyvaskyla, Finland; h Isala Heart Center, Zwolle, the Netherlands; i Sports Medicine Department Isala, Zwolle, the Netherlands; j Institute for Physical Activity and Nutrition, Deakin University, Geelong, Victoria, Australia; k Cardiac Rehabilitation Integrated Practice Unit, Althaia Xarxa Assistencial Universitària de Manresa, Manresa, Spain; l Faculty of Health Sciences at Manresa, Universitat de Vic - Universitat Central de Catalunya (UVic-UCC), Manresa, Spain; m Central Catalonia Chronicity Research Group (C3RG), Institut de Recerca i Innovació en Ciències de la Vida i de la Salut a la Catalunya Central (IRIS-CC), Vic, Spain; n Faculty of Health Sciences, Simon Fraser University, Burnaby, Canada; o Division of Cardiology, Providence Health Care, Vancouver, Canada; p Nursing Department, Université du Québec à Trois-Rivières, Trois-Rivières City, Québec, Canada; q Danish Center for Clinical Health Services Research, Department of Clinical Medicine, Aalborg University, Aalborg, Denmark; r Department of Cardiology, Radboud University Medical Center, Nijmegen, the Netherlands; s Department of Cardiology, Maxima Medical Centre, Veldhoven, the Netherlands; t Robertson Centre for Biostatistics, University of Glasgow, Glasgow, United Kingdom

## 1 Introduction

Angina pectoris (AP) is a common condition in Western society. Contemporary treatment of AP includes optimal medical therapy, which comprises preventive and anti-anginal medication to reduce morbidity and mortality, and improve health-related quality of life (HRQoL). When symptoms persist, revascularisation should be considered. However, trials have questioned the benefits of revascularisation on morbidity and mortality in patients with stable AP [1], highlighting the need to explore other therapeutic strategies. Interestingly, retrospective evidence showed lower risks for morbidity and mortality following ExCR compared to revascularisation [2]. Randomised controlled trials (RCTs) on the contemporary benefits of ExCR in patients with AP are scarce, and are limited by small sample sizes and/or heterogeneity in design [3]. Therefore, we aimed to provide contemporary estimates on the effect of ExCR in patients with AP.

## 2 Methods

This study represents a secondary analysis of The Cardiac Rehabilitation Meta-Analysis of Trials in patients with coronary heart disease (CHD) using individual participant data (CaReMATCH) study. The main results of CaReMATCH have been described elsewhere [4]. The ethics committee of the Radboudumc (Nijmegen, The Netherlands) waived the requirement for ethical approval (2022-15847). Included trials were approved by their local ethics committee.

For the present study, we exclusively selected individuals diagnosed with angina pectoris. RCTs were eligible if they 1) were published since 2010, 2) had a minimum of 6-months follow-up, and 3) compared ExCR to usual care without any structured exercise prescription. ExCR was supervised or non-supervised, either with or without additional psychosocial or educational intervention, and managed in an outpatient, community- or home-based setting. AP was defined as both stable and unstable angina, with most trials being unable to differentiate between the two. The primary outcome was HRQoL, and was measured using one of four validated questionnaires, including the Euro Quality of Life questionnaire (EQ-5D), Short Form 36-item health survey (SF-36), 15-dimensional quality of life questionnaire (15D), and Quality of Life Index – cardiac version. To combine questionnaires, primary analyses on HRQoL were based on calculating standardized scores (i.e., Z-scores). To ease interpretation of HRQoL effect sizes, we performed secondary analyses by pooling data from questionnaires reporting HRQoL as utility indices (UIs).

Secondary outcomes included composite outcomes of 1) all-cause mortality and rehospitalisation, and 2) cardiovascular-related mortality and rehospitalisation. Time to event data were defined as the interval between randomisation and event or right censoring, whichever occurred first. We adopted one-stage linear models as our primary analysis in the intention-to-treat population with complete follow-up data. Linear and Cox mixed-effect regression models were used to quantify the overall effect of ExCR on HRQoL and time-to-event outcomes, respectively. Given the variation in trial follow-up timings, we pooled HRQoL data from the last follow-up timing with a maximum follow-up of 12 months. To evaluate the robustness of our results, we performed: 1) two-stage meta-analyses using random effects, and 2) omitted the largest trial from the analyses (Snoek et al.; **Supplemental References**). Data were presented as means and standard errors (SE), medians (interquartile range (IQR)) or frequencies ( %) as appropriate. Reporting were performed in line with the Preferred Reporting Items for Systematic Review and Meta-Analyses of Individual Participant Data (PRISMA-IPD) statement (**eTable 1**).

## 3 Results

Seven out of eight trials included in CaReMATCH involved individuals with AP (**Supplemental References, eFigure 1, eTable 2**), comprising 286 participants. Baseline characteristics were well-balanced across groups (Table 1). Participants were frequently male (*n* = 229, 80.1% men) and had a mean age of 68±8.9 years. Comorbidities were frequently present (hypertension: *n* = 189, 66.1%, diabetes: *n* = 62, 21.7%). Trials were published between 2012 and 2020, and were frequently performed in Europe (*n* = 4). Delivery of ExCR was often home-based (*n* = 4), or combined with center-based sessions (*n* = 2), with all trials including aerobic exercise (**eTable 2**).

**Table 1:** Baseline characteristics of patients with angina pectoris randomized to either ExCR or non-ExCR controls.

	ExCR (*n* = 143)	Controls (*n* = 143)
Age, years (SE)	68.1 (0.74)	68.3 (0.85)
Sex, n women ( %)	31 (21.7)	26 (18.2)
BMI, kg/m^2^ (SE)	28.1 (0.37)	28.1 (0.41)
LVEF, % (SE)	53 (1.7)	57 (1.5)
Current smoker, n ( %)	12 (8.4)	8 (5.6)
Medication use, n ( %) *Betablockers* *ACE-inhibitors* *ARB* *Antilipids* *Diuretics* *Antithrombotics*	94 (65.7) 64 (44.8) 14 (17.9) 132 (92.3) 45 (31.5) 139 (97.2)	100 (69.9) 68 (47.6) 16 (19.0) 128 (89.5) 29 (20.3) 136 (95.8)
Hypertension, n ( %)	101 (79.5)	88 (68.2)
Dyslipidaemia, n ( %)	103 (81.1)	99 (76.7)
Diabetes, n ( %)	37 (26.1)	25 (17.5)
Revascularisation *PCI, n (%)* *CABG, n (%)*	71 (49.7) 23 (24.2)	72 (61.0) 25 (21.2)
*PCI + CABG, n (%)*	6 (4.2)	4 (2.8)
*No revascularisation, n (%)*	20 (14.0)	26 (18.2)
*Unknown, n (%)*	23 (16.1)	25 (17.5)

Data were presented as means (mean difference (MD)) and standard errors (SE), medians (interquartile range (IQR)) or frequencies ( %) as appropriate. ACE-inhibitors angiotensin-converting enzyme inhibitors, ARB angiotensin receptor blocker, BMI body mass index, ExCR exercise based cardiac rehabilitation, LVEF left ventricular ejection fraction, PCI percutaneous coronary intervention, CABG coronary artery bypass graft.

**Fig. 1:**
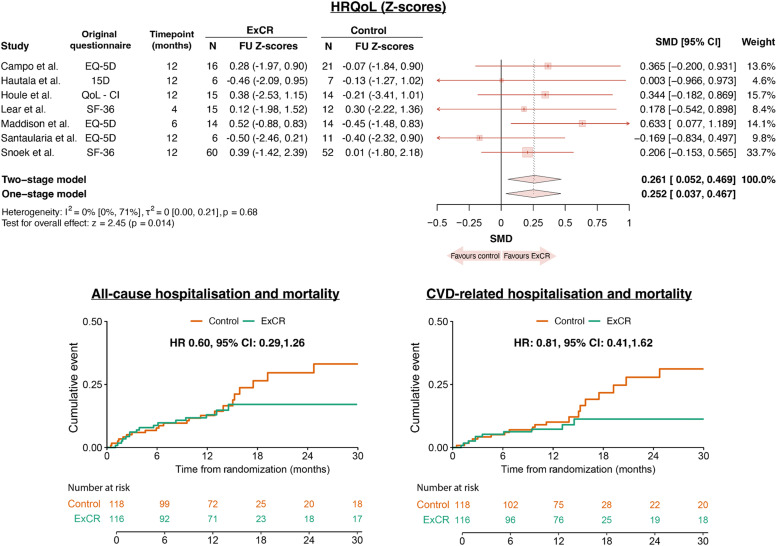
**Effect of ExCR on HRQoL and time-to-event outcomes.** Multipanel figure presenting the effect of participation in ExCR on HRQoL (upper panel) and all-cause and cardiovascular-related hospitalisation and mortality (bottom panels). Compared to non-ExCR controls, participation in ExCR resulted in an increased HRQoL up to 12 months (SMD 0.252 [95% CI: 0.037, 0.467]). Additionally, participation in ExCR did not reduce the risk of all-cause or CVD-related hospitalisation and mortality (HR 0.60, 95% CI 0.29, 1.26; HR 0.81, 95% CI: 0.41, 1.62, respectively), although 95% CI were wide. Results were derived from linear and Cox mixed effects models respectively. Given the variation in trial follow-up timings, we pooled HRQoL data from the last follow-up timing with a maximum follow-up of 12 months. All models on HRQoL were corrected for the baseline HRQoL. HRQoL was expressed as standardized scores (i.e. Z-scores). CI confidence interval, CVD cardiovascular disease, EQ-5D Euro Quality of Life with 5 dimensions, ExCR exercise-based cardiac rehabilitation, FU follow-up, HRQoL health-related quality of life, HR hazard ratio, QoL CI quality of life – cardiac index, SF-36 Short Form 36-item health survey, SMD standardized mean difference, UI utility index, 15D 15-dimensional quality of life questionnaire.

Compared to controls, participation in ExCR significantly increased HRQoL measured as Z-scores (standardised mean difference (SMD): 0.252, 95% confidence interval (CI): 0.037 to 0.467) (Fig. 1*)*. Comparable inferences were made when pooling questionnaires reporting UIs, although 95%CI’s were wider (mean difference (MD): 0.031, 95% CI: −0.004, 0.067) (**eFigure 2**). During a median follow-up of 12.5 months (IQR: 11.3, 17.6), 38 events occurred related to mortality or hospitalisation (ExCR: *n* = 16, control: *n* = 22), with 29 events being cardiovascular-related (ExCR: *n* = 10, control: *n* = 19). Participation in ExCR did not significantly reduce the risk for all-cause (HR 0.60, 95% CI; 0.29 to 1.26) or cardiovascular-related mortality and hospitalisation (HR: 0.81, 95% CI; 0.41 to 1.62, **Figure 2**). Observations were consistent across secondary analyses (**eTable 2, eFigure 3–4**).

## 4 Discussion

In patients with AP, participation in ExCR significantly improved HRQoL compared to non-ExCR controls up to 12-month follow-up. The 2018 Cochrane review found no beneficial effects of ExCR on HRQoL compared to non-ExCR controls [3]. Importantly, six out of the seven RCTs included in the 2018 Cochrane-review were published more than two decades ago. This may be explained by the observation that studies published in the past decade typically included a case-mix of patients with CHD rather than a specified population of AP. Pooling IPD from multiple trials bypasses this limitation, as we exclusively included trials conducted since 2010 and selected participants with AP only. Consequently, this led to a sample size of 286 participants that is substantially larger than previous RCTs, which varied between 24 and 113 participants [3]. This enabled us to demonstrate that contemporary ExCR significantly improved HRQoL in patients with AP. This observation has significant clinical importance, especially since improving patients’ HRQoL is one of the main goals in the treatment of chronic coronary syndromes [5].

Following our secondary aim, we did not observe significant effects of ExCR on all-cause or cardiovascular-related mortality or hospitalisation risk across 12-month follow-up. Potential explanations for this observation include the relatively low event rate in patients with AP, combined with the relatively small sample size and short follow-up. Supporting this, effect sizes of ExCR on all-cause and cardiovascular-related hospitalisation and mortality observed in our IPD meta-analysis are comparable to those presented in meta-analyses on the effects of ExCR in patients with heart failure or CHD ([6,7]). Therefore, ExCR may have the potential to reduce mortality and hospitalisation risk in patients with AP, although further research using a sufficiently powered sample size is needed.

Our study has some limitations. First, we were unable to distinguish between patients with stable and unstable AP in several included trials. Since the majority of included trials excluded conditions that interfere with performing physical activity, we expect that the majority of patients included stable AP. Second, no data on timing of revascularisation prior to ExCR was available, so we could not evaluate the clinically relevant question whether the timing of ExCR affected our outcomes. Future studies are recommended to specifically focus on the timing of ExCR in relation to the revascularisation procedure, as one may adopt ExCR as a post-revascularisation treatment, but also as a first-line approach prior to potential need for revascularisation.

## 5 Conclusion

This CaReMATCH IPD meta-analysis found contemporary ExCR participation to significantly improve HRQoL in patients with AP, although we did not observe significant effects on all-cause and cardiovascular-related mortality and hospitalisations over a 12-month follow-up. The improvement in HRQoL confirms the benefit of participating in ExCR, highlighting the need to further study the potential wider clinical effects of ExCR in patients with AP.

## Authorship agreement

All co-authors were involved in the interpretation of data, co-writing/critically revising the manuscript, approved its final form and agree with submission to *American Journal of Preventive Cardiology*. The manuscript has not been published and is not being considered for publication elsewhere in whole or part in any language. We declare to be accountable for all aspects of the work and questions related to accuracy or integrity of any part of the work will be appropriately investigated and resolved.

## Funding

This project was financially supported by the Radboud-Glasgow Collaboration Fund. The sponsor had no role in the design, data handling and analysis, interpretation of results and reporting of this study.

## Disclosures

None to declare.

## Statement of authorship (CRediT)

IdK, JMH, BJRB, GOD, GYHL, RJMvG, TV, HMCK, RST, NAS and DHJT conceptualized and designed the study. NAS and BJRB wrote the protocols, with feedback from IdK, JMH, GOD, GYHL, RST, and DHJT. GC, AJH, JAS, RM, NS, SAL and JH acquired and contributed individual participant data from their respective trial. Formal analyses, programming of code, data curation and visualization of results were performed by NAS. Validation of data were performed by IdK, JMH and NAS. Interpretation of results were performed by IdK, JMH, BJRB, GOD, RST, NAS and DHJT. The first version of the manuscript was drafted by IdK and JMH, and feedback was provided by all other authors (BJRB, GOD, GC, AJH, JAS, RM, NS, SAL, JH, GYHL, RJMvG, TV, HMCK, RST, NAS, DHJT). All authors have approved the final manuscript. Those involved in the CaReMATCH collaborators were involved in the design and conduct of the respective trial included in CaReMATCH, and have been listed in the Supplemental Material.

## CRediT authorship contribution statement

**Iris A. De Koning:** Writing – original draft, Validation, Project administration, Methodology, Conceptualization. **Joyce.M. Heutinck:** Writing – original draft, Validation, Project administration, Methodology, Conceptualization. **Benjamin J.R. Buckley:** Writing – review & editing, Methodology, Conceptualization. **Grace O. Dibben:** Writing – review & editing, Methodology, Conceptualization. **Gianluca Campo:** Writing – review & editing, Investigation. **Arto J. Hautala:** Writing – review & editing, Investigation. **Johan A. Snoek:** Writing – review & editing, Investigation. **Ralph Maddison:** Writing – review & editing, Investigation. **Núria Santaularia:** Writing – review & editing, Investigation. **Scott A. Lear:** Writing – review & editing, Investigation. **Julie Houle:** Writing – review & editing, Investigation. **Gregory Y.H. Lip:** Writing – review & editing, Methodology, Conceptualization. **Robert Jan M. Van Geuns:** Writing – review & editing, Methodology, Conceptualization. **Tom Vromen:** Writing – review & editing, Methodology, Conceptualization. **Hareld M.C. Kemps:** Writing – review & editing, Methodology, Conceptualization. **Rod S. Taylor:** Writing – review & editing, Supervision, Methodology, Funding acquisition, Conceptualization. **Niels A. Stens:** Writing – review & editing, Visualization, Validation, Software, Project administration, Methodology, Formal analysis, Data curation, Conceptualization. **Dick H.J. Thijssen:** Writing – review & editing, Supervision, Methodology, Funding acquisition, Conceptualization.

## Declaration of competing interest

None to declare.

## Appendix Supplementary materials

Image, application 1
