# Supplementary material for: Exercise-based cardiac rehabilitation for patients with angina pectoris - a CaReMATCH individual participant data meta-analysis
Source: Am J Prev Cardiol. 2026 Mar 10;28:101535. doi: 10.1016/j.ajpc.2026.101535 (PMC13325976; doi:10.1016/j.ajpc.2026.101535)
Supplement: Supplementary file 1 [file mmc1.docx]

**Exercise-based Cardiac Rehabilitation for patients with Angina Pectoris**

*A CaReMATCH Individual Participant Data Meta-analysis*

**Supplemental material**

[**CaReMATCH collaborators** 2](#_Toc223690884)

[**Supplemental References. References of included trials.** 4](#_Toc223690885)

[**eFigure 1. PRISMA flowchart of search results and study selection.** 6](#_Toc223690886)

[**eFigure 2. Effect of exercise-based cardiac rehabilitation on HRQoL up to 12 months of follow-up, expressed as utility indices.** 7](#_Toc223690887)

[**eFigure 3. The effect of ExCR on HRQoL up to 12 months of follow-up, excluding Snoek et al.** 8](#_Toc223690888)

[**eFigure 4. Pooling effects of ExCR on all-cause and cardiovascular-related mortality and hospitalisation using two-stage models.** 9](#_Toc223690889)

[**eTable 1. PRISMA-IPD Checklist of items to include when reporting a systematic review and meta-analysis of individual participant data (IPD)** 10](#_Toc223690890)

[**eTable 2. Trial-level characteristics of the seven trials included in the CaReMATCH study that included patients with angina pectoris.** 14](#_Toc223690891)

[**eTable 3. Baseline characteristics of patients with angina pectoris randomized to either ExCR or non-ExCR controls, Snoek et al. excluded.** 16](#_Toc223690892)

[**References** 17](#_Toc223690893)

# **CaReMATCH collaborators**

| **Name** | **Affiliations** |
| --- | --- |
| Alun D. Hughes, MD | - MRC Unit for Lifelong Health and Ageing at University College London, London, United Kingdom |
| Ajay S. Vamadevan, PhD | - Centre for Chronic Disease Control, New Delhi, India - Public Health Foundation of India, New Delhi, India |
| Ambalam M Chandrasekaran, MSc | - Centre for Chronic Disease Control, Public Health Foundation India, New Delhi, India. |
| Ambuj Roy, MD, DM | - All India Institute of Medical Sciences, New Delhi, India |
| Andrea Raisi, MSc | - Center for Exercise Science and Sport, University of Ferrara, Ferrara, Italy. |
| Anil R Jain, MS | - SAL Hospital and Medical Institute, Ahmedabad, India |
| Arnoud W.J. van’t Hof, MD, PhD | - Department of Cardiology, Maastricht University Medical Center and Cardiovascular Research Institute Maastricht (CARIM), Maastricht, the Netherlands |
| Bhaskara Rao, MD | - CARE Hospital Heart institute, Vishakapatnam, India |
| Bishav Mohan, MD | - Dayanand Medical College, Ludhiana, India |
| Davinder S Chadha, MD | - Command Hospital, Bengaluru, India |
| Dimple Kondal, PhD | - Centre for Chronic Disease Control, New Delhi, India |
| Divya Soni, MSc | - Centre for Chronic Disease Control, New Delhi, India |
| Dorairaj Prabhakaran, MD, DM | - Centre for Chronic Disease Control, Public Health Foundation India, New Delhi, India. - Department of Non-communicable Disease Epidemiology, London School of Hygiene and Tropical Medicine, London, UK |
| Ed P. de Kluiver, PhD | - None |
| Elisabetta Tonet, MD | - Cardiology Unit, Azienda Ospedaliera Universitaria di Ferrara, Ferrara, Italy |
| Gianni Mazzoni, MD | - Center for Exercise Science and Sport, University of Ferrara, Ferrara, Italy. |
| Giovanni Grazzi, MD | - Center for Exercise Science and Sport, University of Ferrara, Ferrara, Italy. |
| Ian Roberts, PhD | - London School of Hygiene and Tropical Medicine, London, United Kingdom |
| Jabir Abdullakutty, DM | - Lisie Hospital, Kochi, India |
| Kalpana Singh, PhD | - Centre for Chronic Disease Control, New Delhi, India |
| Kaushik Chattopadhyay, MSc | - London School of Hygiene and Tropical Medicine, London, United Kingdom - University of Nottingham, Nottingham, United Kingdom. |
| Kavita Singh, PhD | - Centre for Chronic Disease Control, New Delhi, India |
| Kolli S Reddy, DM | - Public Health Foundation of India, New Delhi, India |
| Kushal Madan, PhD | - Sir Ganga Ram Hospital, New Delhi, India |
| Manjunath C Nanjappa, DM | - Sri Jayadeva Institute of Cardiovascular Sciences and Research, Bengaluru, India |
| Nagamalesh U Madappa, DM | - M S Ramaiah Medical College and Hospital, Bengaluru, India |
| Nagraj Desai, DM | - JSS Medical College, Mysuru, India |
| Narsimhan Calambur, DM | - CARE Hospital, Hyderabad, India |
| Natrajan KU, DM | - Amrita Institute of Medical Sciences, Kochi, India |
| Nikhil Tandon, DM | - All India Institute of Medical Sciences, New Delhi, India |
| Nishi Chathurvedi, MD | - MRC Unit for Lifelong Health and Ageing at University College London, London, United Kingdom - Imperial College London, London, United Kingdom. |
| Paul Poirier, MD, PhD | - Faculty of Pharmacy, Université Laval - Institut universitaire de cardiologie et de pneumologie de Québec |
| Poppy Malinson, MSc | - London School of Hygiene and Tropical Medicine, London, United Kingdom. |
| Prabhavathi Bhat, DM | - Sri Jayadeva Institute of Cardiovascular Sciences and Research, Bengaluru, India. |
| Pradeep A Praveen, PhD | - Centre for Chronic Disease Control, New Delhi, India - All India Institute of Medical Sciences, New Delhi, India |
| Prakash C Negi, DM | - Indira Gandhi Medical College, Shimla, India |
| Prasad MR, DM | - KLE Academy of Higher Education & Research/ College/university, Belgaum, India |
| Raghava Sarma, DM | - Lalitha Super Specialities Hospital, Kothapeta, India |
| Raji Devarajan, MSc | - Centre for Chronic Disease Control, New Delhi, India. |
| Ravindra K Tongia, DM | - Escorts Fortis Hospital, Jaipur, India |
| Rita Pavasini, MD | - Cardiology Unit, Azienda Ospedaliera Universitaria di Ferrara, Ferrara, Italy. |
| Sadananda S Kanchanahalli, DM | - Sri Jayadeva Institute of Cardiovascular Sciences and Research, Mysuru, India |
| Sanjay Kinra, MD, PhD | - Department of Non-communicable Disease Epidemiology, London School of Hygiene and Tropical Medicine, London, UK |
| Satish Patil, MD | - SRI BM.Patil medical college, Vijayapura, India. |
| Shah Ebrahim, MD | - London School of Hygiene and Tropical Medicine, London, United Kingdom. |
| Shankar Patil, MD | - SRI BM.Patil medical college, Vijayapura, India. |
| Sharad Chandra, DM | - King George’s Medical University,  Lucknow, India |
| Srikumar Swaminathan, DM | - Frontier Lifeline Hospital, Chennai, India |
| Srinivas Mallya, MS | - SAL Hospital and Medical Institute, Ahmedabad, India |
| Stefano Volpato, MD, MPH | - Department of Medical Science, University of Ferrara, Ferrara, Italy |
| Stuart Pocock, PhD | - London School of Hygiene and Tropical Medicine, London, United Kingdom |
| Subhash C Manchanda, DM | - Sir Ganga Ram Hospital, New Delhi, India |
| Subramaniam Natarajan, DM | - G.Kuppuswamy Naidu Memorial Hospital, Coimbatore, India |
| Sudhir R Naik, DM | - Apollo Hospital, Jubilee Hills, Hyderabad, India |
| Sunil Kumar, DM | - JSS Hospital, Mysuru, India |

#

# **Supplemental References. References of included trials.**

*EFEX-CARE, 2017*

- Hautala AJ, Kiviniemi AM, Mäkikallio T, et al. Economic evaluation of exercise-based cardiac rehabilitation in patients with a recent acute coronary syndrome. Scand J Med Sci Sports. Nov 2017;27(11):1395-1403. doi:10.1111/sms.12738
- Hautala AJ, Shavazipour B, Afsar B, Tulppo MP, Miettinen K. Machine learning models for assessing risk factors affecting health care costs: 12-month exercise-based cardiac rehabilitation. Front Public Health. 2024;12:1378349. doi:10.3389/fpubh.2024.1378349

*EU-CaRE, 2021*

- Prescott E, Meindersma EP, van der Velde AE, et al. A EUropean study on effectiveness and sustainability of current Cardiac Rehabilitation programmes in the Elderly: Design of the EU-CaRE randomised controlled trial. Eur J Prev Cardiol. Oct 2016;23(2 suppl):27-40. doi:10.1177/2047487316670063
- Snoek JA, Prescott EI, van der Velde AE, et al. Effectiveness of Home-Based Mobile Guided Cardiac Rehabilitation as Alternative Strategy for Nonparticipation in Clinic-Based Cardiac Rehabilitation Among Elderly Patients in Europe: A Randomized Clinical Trial. JAMA Cardiol. Apr 1 2021;6(4):463-468. doi:10.1001/jamacardio.2020.521

*HEART, 2015*

- Maddison R, Whittaker R, Stewart R, et al. HEART: heart exercise and remote technologies: a randomized controlled trial study protocol. BMC Cardiovasc Disord. May 31 2011;11:26. doi:10.1186/1471-2261-11-26
- Maddison R, Pfaeffli L, Stewart R, et al. The HEART Mobile Phone Trial: The Partial Mediating Effects of Self-Efficacy on Physical Activity among Cardiac Patients. Front Public Health. 2014;2:56. doi:10.3389/fpubh.2014.00056
- Maddison R, Pfaeffli L, Whittaker R, et al. A mobile phone intervention increases physical activity in people with cardiovascular disease: Results from the HEART randomized controlled trial. Eur J Prev Cardiol. Jun 2015;22(6):701-9. doi:10.1177/2047487314535076

*Houle et al., 2012*

- Houle J, Doyon O, Vadeboncoeur N, Turbide G, Diaz A, Poirier P. Effectiveness of a pedometer-based program using a socio-cognitive intervention on physical activity and quality of life in a setting of cardiac rehabilitation. Can J Cardiol. Jan-Feb 2012;28(1):27-32. doi:10.1016/j.cjca.2011.09.020

*HULK, 2020*

- Tonet E, Maietti E, Chiaranda G, et al. Physical activity intervention for elderly patients with reduced physical performance after acute coronary syndrome (HULK study): rationale and design of a randomized clinical trial. BMC Cardiovasc Disord. May 21 2018;18(1):98. doi:10.1186/s12872-018-0839-8
- Campo G, Tonet E, Chiaranda G, et al. Exercise Intervention to Improve Functional Capacity in Older Adults After Acute Coronary Syndrome. J Am Coll Cardiol. Dec 10 2019;74(23):2948-2950. doi:10.1016/j.jacc.2019.10.010
- Campo G, Tonet E, Chiaranda G, et al. Exercise intervention improves quality of life in older adults after myocardial infarction: randomised clinical trial. Heart. Nov 2020;106(21):1658-1664. doi:10.1136/heartjnl-2019-316349

*Lear et al., 2014*

- Lear SA, Singer J, Banner-Lukaris D, et al. Randomized trial of a virtual cardiac rehabilitation program delivered at a distance via the Internet. Circ Cardiovasc Qual Outcomes. Nov 2014;7(6):952-9. doi:10.1161/circoutcomes.114.001230
- Lear SA, Singer J, Banner-Lukaris D, et al. Improving access to cardiac rehabilitation using the internet: a randomized trial. Stud Health Technol Inform. 2015;209:58-66.
- Mendell J, Bates J, Banner-Lukaris D, et al. What Do Patients Talk About? A Qualitative Analysis of Online Chat Sessions with Health Care Specialists During a "Virtual" Cardiac Rehabilitation Program. Telemed J E Health. Jan 2019;25(1):71-78. doi:10.1089/tmj.2017.0206

*Santaularia et al., 2017*

- Santaularia N, Caminal J, Arnau A, et al. Randomized clinical trial to evaluate the effect of a supervised exercise training program on readmissions in patients with myocardial ischemia: a study protocol. BMC Cardiovasc Disord. Apr 25 2013;13:32. doi:10.1186/1471-2261-13-32
- Santaularia N, Caminal J, Arnau A, et al. The efficacy of a supervised exercise training programme on readmission rates in patients with myocardial ischemia: results from a randomised controlled trial. Eur J Cardiovasc Nurs. Mar 2017;16(3):201-212. doi:10.1177/1474515116648801
- Santaularia N, Arnau A, Jaarsma T, Torà N, Vázquez-Oliva G. Efficacy of a supervised exercise training program on five-year readmission rates in patients with acute coronary syndrome. A randomised controlled trial. Rehabilitacion (Madr). Jan-Mar 2023;57(1):100720. doi:10.1016/j.rh.2021.12.001

#
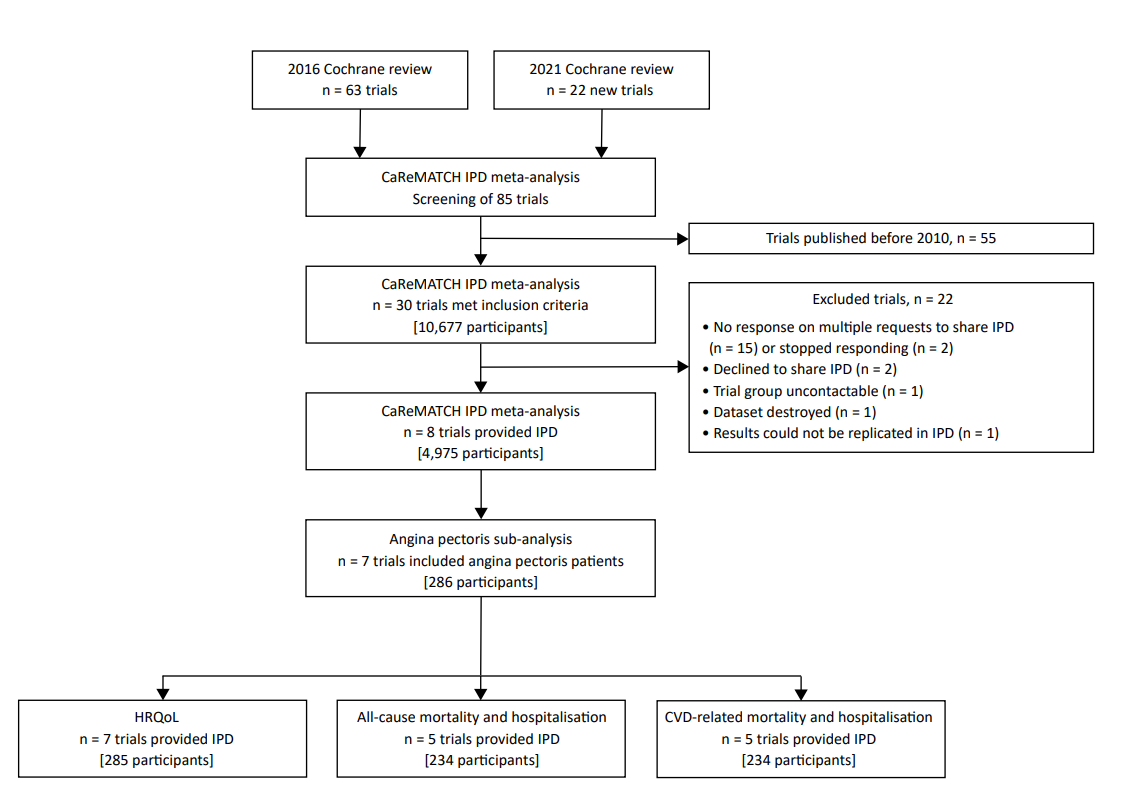
**eFigure 1. PRISMA flowchart of search results and study selection.**

*Out of 85 trials, 30 trials (n: 10,677) were eligible. IPD from 7 trials were analysed, comprising 286 participants. CVD cardiovascular disease, HRQoL health-related quality of life, IPD individual participant data.*

# **
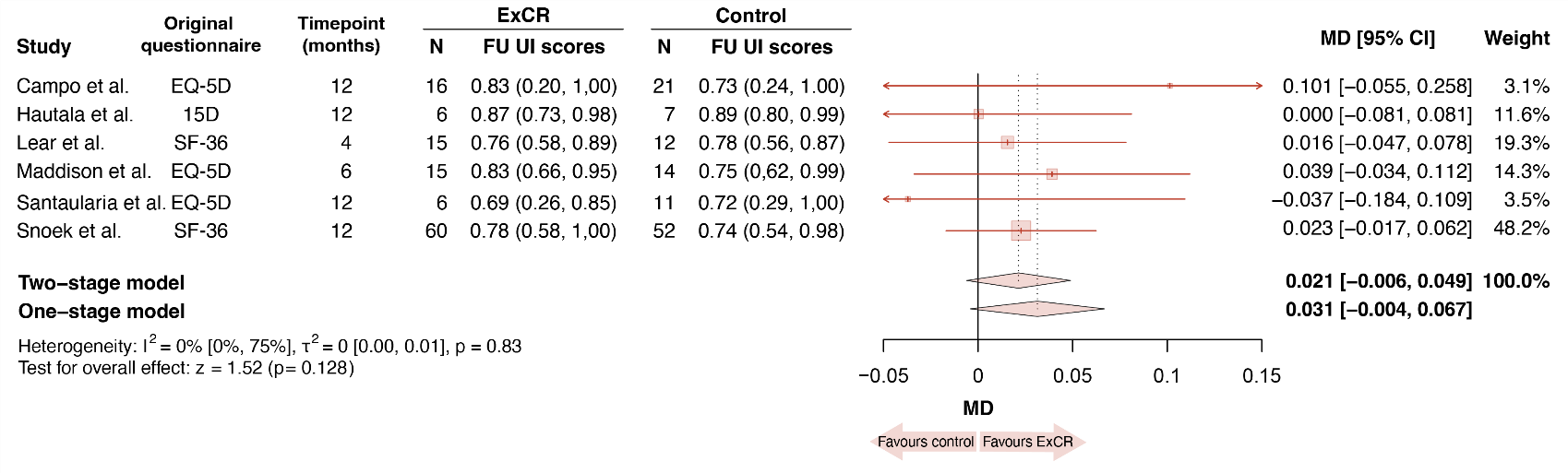
eFigure 2. Effect of exercise-based cardiac rehabilitation on HRQoL up to 12 months of follow-up, expressed as utility indices.**

*Forest plot highlighting that participation likely increases HRQol compared to controls, although 95% confidence intervals were wide. Given the variation in trial follow-up timings, we pooled HRQoL data from the last follow-up timing with a maximum follow-up of 12 months. All models were corrected for the baseline HRQoL. For each study, red horizontal lines represent the effect estimate and 95% confidence interval. Study weights were obtained via a random-effects meta-analysis and are presented as shaded squares and percentages. The red diamond represents the pooled estimate and its 95% confidence interval. CI confidence interval, EQ-5D Euro Quality of Life with 5 dimensions, ExCR exercise-based cardiac rehabilitation, FU follow-up, HRQoL health-related quality of life, MD mean difference, SF-36 Short Form 36-item.*

#
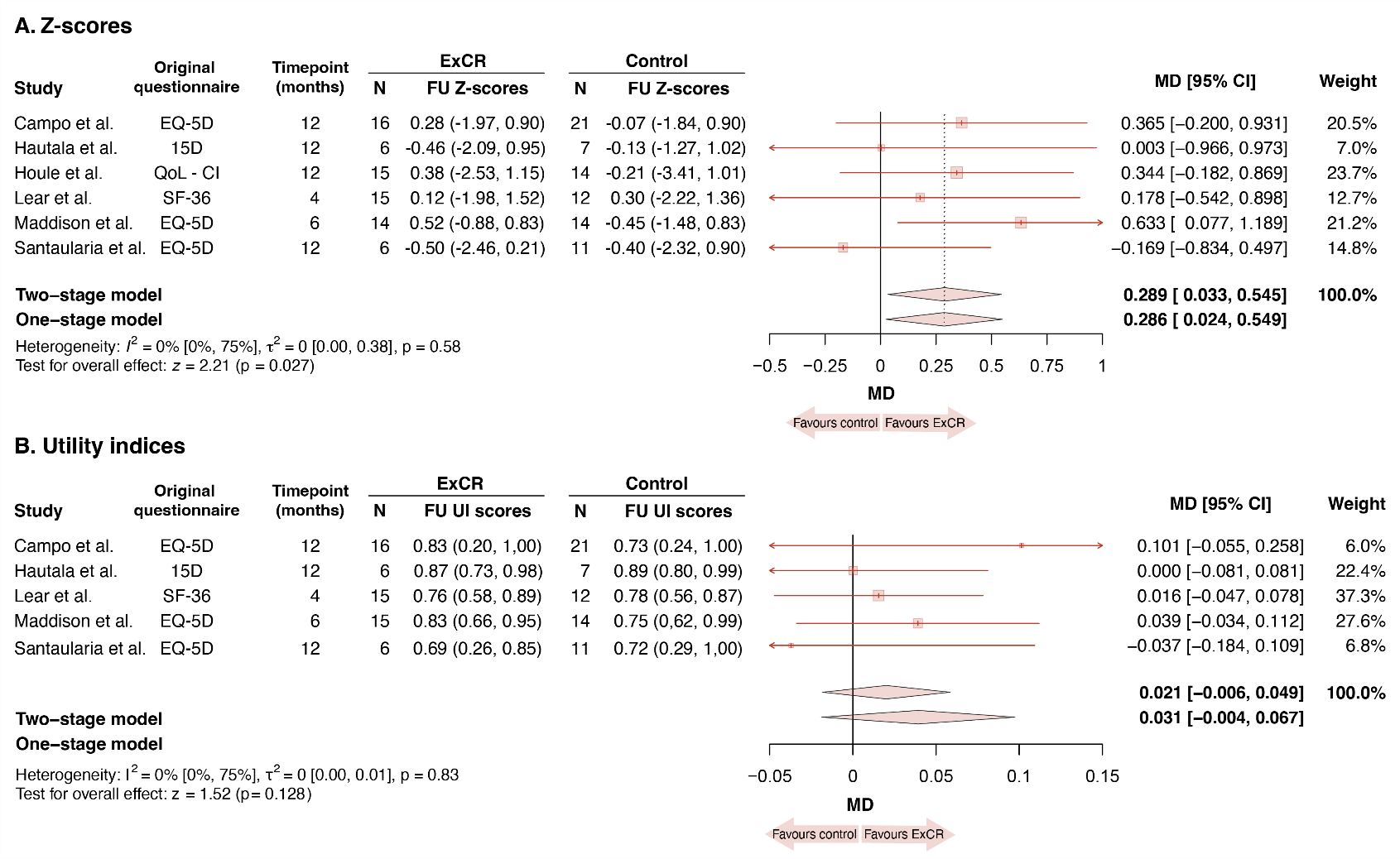
**eFigure 3. The effect of ExCR on HRQoL up to 12 months of follow-up, excluding Snoek et al.**

*Forest plot highlighting that, after excluding Snoek et al., participation in ExCR increased HRQol compared to controls. The forest plot is stratified for HRQoL as expressed by Z-scores (panel A) and UI-scores (panel B). Given the variation in trial follow-up timings, we pooled HRQoL data from the last follow-up timing with a maximum follow-up of 12 months. All models were corrected for the baseline HRQoL. For each study, red horizontal lines represent the effect estimate and 95% confidence interval. Study weights were obtained via a random-effects meta-analysis and are presented as shaded squares and percentages. The red diamond represents the pooled estimate and its 95% confidence interval. CI confidence interval, EQ-5D Euro Quality of Life with 5 dimensions, ExCR exercise-based cardiac rehabilitation, FU follow-up, HRQoL health-related quality of life, MD mean difference, SF-36 Short Form 36-item, UI utility index..*

# **eFigure 4. Pooling effects of ExCR on all-cause and cardiovascular-related mortality and hospitalisation using two-stage models.**

**
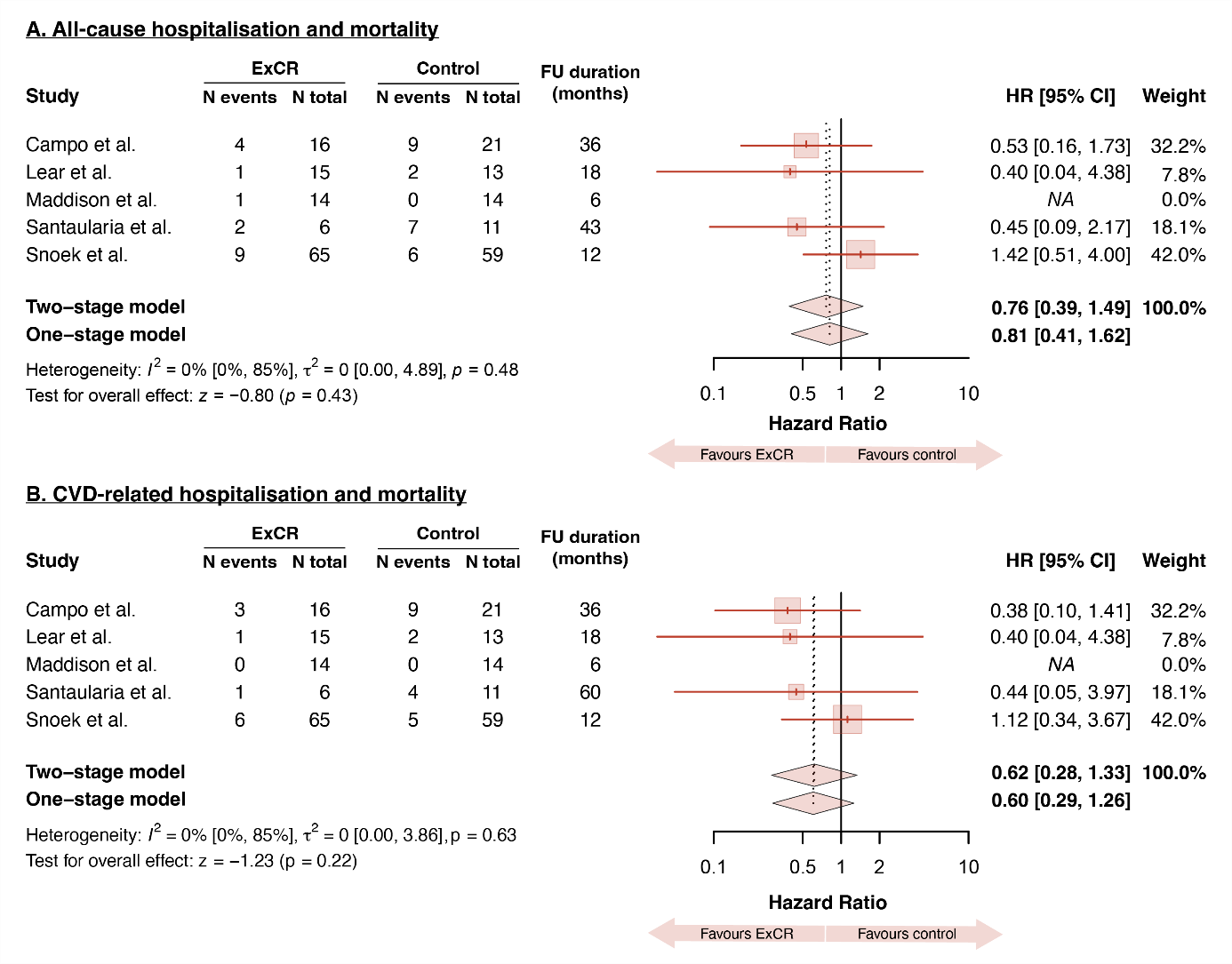
**

*Forest plots on the effects of ExCR on all-cause (panel A) and CVD-related (panel B) hospitalisation and mortality. Effects were pooled using a two-stage framework and are presented alongside pooled effects from one-stage models as documented in our main results. In the two-stage framework, a Cox proportional hazards regression model was applied to each trial individually, and resulting ln-transformed hazard ratios were pooled in a random effects meta-analysis. As hazard ratios cannot reliably be computed in studies with zero events in one or both treatment groups, effect sizes for some individual trials could not be determined using two-stage models. For each study, red horizontal lines represent the effect estimate and 95% CI. Study weights were obtained via a random-effects meta-analysis and are presented as shaded squares and percentages. The red diamond represents the pooled estimate and its 95% CI. Heterogeneity and tests for the overall effect belong to the two-stage models. CI confidence interval, ExCR exercise-based cardiac rehabilitation, FU follow-up, HR hazard ratio, NA not applicable.*

# **eTable 1. PRISMA-IPD Checklist of items to include when reporting a systematic review and meta-analysis of individual participant data (IPD)**

| **PRISMA-IPD**  **Section/topic** | **Item No** | **Checklist item** | **Reported on page** |  |
| --- | --- | --- | --- | --- |
| **Title** | | | | |
| Title | 1 | Identify the report as a systematic review and meta-analysis of individual participant data. | Page 1 |  |
| Abstract | | | | |
| Structured summary | 2 | Provide a structured summary including as applicable: | NA |  |
|  |  | **Background**: state research question and main objectives, with information on participants, interventions, comparators and outcomes. |  |  |
|  |  | **Methods**: report eligibility criteria; data sources including dates of last bibliographic search or elicitation, noting that IPD were sought; methods of assessing risk of bias. |  |  |
|  |  | **Results**: provide number and type of studies and participants identified and number (%) obtained; summary effect estimates for main outcomes (benefits and harms) with confidence intervals and measures of statistical heterogeneity. Describe the direction and size of summary effects in terms meaningful to those who would put findings into practice. |  |  |
|  |  | **Discussion:** state main strengths and limitations of the evidence, general interpretation of the results and any important implications. |  |  |
|  |  | **Other:** report primary funding source, registration number and registry name for the systematic review and IPD meta-analysis. |  |  |
| Introduction | | | | |
| Rationale | 3 | Describe the rationale for the review in the context of what is already known. | Page 4 |  |
| Objectives | 4 | Provide an explicit statement of the questions being addressed with reference, as applicable, to participants, interventions, comparisons, outcomes and study design (PICOS). Include any hypotheses that relate to particular types of participant-level subgroups. | Pages 4 |  |
| Methods | | | | |
| Protocol and registration | 5 | Indicate if a protocol exists and where it can be accessed. If available, provide registration information including registration number and registry name. Provide publication details, if applicable. | Main paper of CaReMATCH |  |
| Eligibility criteria | 6 | Specify inclusion and exclusion criteria including those relating to participants, interventions, comparisons, outcomes, study design and characteristics (e.g. years when conducted, required minimum follow-up). Note whether these were applied at the study or individual level i.e. whether eligible participants were included (and ineligible participants excluded) from a study that included a wider population than specified by the review inclusion criteria. The rationale for criteria should be stated. | Page 4, eFigure 1  Main paper of CaReMATCH |  |
| Identifying studies - information sources | 7 | Describe all methods of identifying published and unpublished studies including, as applicable: which bibliographic databases were searched with dates of coverage; details of any hand searching including of conference proceedings; use of study registers and agency or company databases; contact with the original research team and experts in the field; open adverts and surveys. Give the date of last search or elicitation. | eFigure 1  Main paper of CaReMATCH |  |
| Identifying studies - search | 8 | Present the full electronic search strategy for at least one database, including any limits used, such that it could be repeated. | Main paper of CaReMATCH  2016 and 2021 Cochrane review |  |
| Study selection processes | 9 | State the process for determining which studies were eligible for inclusion. | Page 4  Main paper of CaReMATCH  2016 and 2021 Cochrane review |  |
| Data collection processes | 10 | Describe how IPD were requested, collected and managed, including any processes for querying and confirming data with investigators. If IPD were not sought from any eligible study, the reason for this should be stated (for each such study). | Main paper |  |
|  |  | If applicable, describe how any studies for which IPD were not available were dealt with. This should include whether, how and what aggregate data were sought or extracted from study reports and publications (such as extracting data independently in duplicate) and any processes for obtaining and confirming these data with investigators. |  |  |
| Data items | 11 | Describe how the information and variables to be collected were chosen. List and define all study level and participant level data that were sought, including baseline and follow-up information. If applicable, describe methods of standardizing or translating variables within the IPD datasets to ensure common scales or measurements across studies. | Main paper |  |
| IPD integrity | A1 | Describe what aspects of IPD were subject to data checking (such as sequence generation, data consistency and completeness, baseline imbalance) and how this was done. | Main paper |  |
| Risk of bias assessment in individual studies. | 12 | Describe methods used to assess risk of bias in the individual studies and whether this was applied separately for each outcome. If applicable, describe how findings of IPD checking were used to inform the assessment. Report if and how risk of bias assessment was used in any data synthesis. | Main paper |  |
| Specification of outcomes and effect measures | 13 | State all treatment comparisons of interests. State all outcomes addressed and define them in detail. State whether they were pre-specified for the review and, if applicable, whether they were primary/main or secondary/additional outcomes. Give the principal measures of effect (such as risk ratio, hazard ratio, difference in means) used for each outcome. | Pages 4-5 |  |
| Synthesis methods | 14 | Describe the meta-analysis methods used to synthesize IPD. Specify any statistical methods and models used. Issues should include (but are not restricted to):   - Use of a one-stage or two-stage approach. - How effect estimates were generated separately within each study and combined across studies (where applicable). - Specification of one-stage models (where applicable) including how clustering of participants within studies was accounted for. - Use of fixed or random effects models and any other model assumptions, such as proportional hazards. - How (summary) survival curves were generated (where applicable). - Methods for quantifying statistical heterogeneity (such as I^2^ and τ^2^). - How studies providing IPD and not providing IPD were analyzed together (where applicable). - How missing data within the IPD were dealt with (where applicable). | Page 5  Main paper of CaReMATCH |  |
| Exploration of variation in effects | A2 | If applicable, describe any methods used to explore variation in effects by study or participant level characteristics (such as estimation of interactions between effect and covariates). State all participant-level characteristics that were analyzed as potential effect modifiers, and whether these were pre-specified. | Main paper of CaReMATCH |  |
| Risk of bias across studies | 15 | Specify any assessment of risk of bias relating to the accumulated body of evidence, including any pertaining to not obtaining IPD for particular studies, outcomes or other variables. | Main paper of CaReMATCH  2016 and 2021 Cochrane reviews |  |
| Additional analyses | 16 | Describe methods of any additional analyses, including sensitivity analyses. State which of these were pre-specified. | Page 5, Figure 1, eFigures 2-4, eTable 3 |  |
| Results | | | | |
| Study selection and IPD obtained | 17 | Give numbers of studies screened, assessed for eligibility, and included in the systematic review with reasons for exclusions at each stage. Indicate the number of studies and participants for which IPD were sought and for which IPD were obtained. For those studies where IPD were not available, give the numbers of studies and participants for which aggregate data were available. Report reasons for non-availability of IPD. Include a flow diagram. | eFigure 1 |  |
| Study characteristics | 18 | For each study, present information on key study and participant characteristics (such as description of interventions, numbers of participants, demographic data, unavailability of outcomes, funding source, and if applicable duration of follow-up). Provide (main) citations for each study. Where applicable, also report similar study characteristics for any studies not providing IPD. | eTable 2 |  |
| IPD integrity | A3 | Report any important issues identified in checking IPD or state that there were none. | Main paper of CaReMATCH |  |
| Risk of bias within studies | 19 | Present data on risk of bias assessments. If applicable, describe whether data checking led to the up-weighting or down-weighting of these assessments. Consider how any potential bias impacts on the robustness of meta-analysis conclusions. | Main paper of CaReMATCH |  |
| Results of individual studies | 20 | For each comparison and for each main outcome (benefit or harm), for each individual study report the number of eligible participants for which data were obtained and show simple summary data for each intervention group (including, where applicable, the number of events), effect estimates and confidence intervals. These may be tabulated or included on a forest plot. | Figure 1 |  |
| Results of syntheses | 21 | Present summary effects for each meta-analysis undertaken, including confidence intervals and measures of statistical heterogeneity. State whether the analysis was pre-specified, and report the numbers of studies and participants and, where applicable, the number of events on which it is based. | Figure 1 |  |
|  |  | When exploring variation in effects due to participant or study characteristics, present summary interaction estimates for each characteristic examined, including confidence intervals and measures of statistical heterogeneity. State whether the analysis was pre-specified. State whether any interaction is consistent across trials. | Main paper of CaReMATCH |  |
|  |  | Provide a description of the direction and size of effect in terms meaningful to those who would put findings into practice. | Pages 5-7 |  |
| Risk of bias across studies | 22 | Present results of any assessment of risk of bias relating to the accumulated body of evidence, including any pertaining to the availability and representativeness of available studies, outcomes or other variables. | Main paper of CaReMATCH |  |
| Additional analyses | 23 | Give results of any additional analyses (e.g. sensitivity analyses). If applicable, this should also include any analyses that incorporate aggregate data for studies that do not have IPD. If applicable, summarize the main meta-analysis results following the inclusion or exclusion of studies for which IPD were not available. | Page 6, Figure 1, eFigures 2-4, eTable 3 |  |
| Discussion | | | | |
| Summary of evidence | 24 | Summarize the main findings, including the strength of evidence for each main outcome. | Page 6 |  |
| Strengths and limitations | 25 | Discuss any important strengths and limitations of the evidence including the benefits of access to IPD and any limitations arising from IPD that were not available. | Page 6 |  |
| Conclusions | 26 | Provide a general interpretation of the findings in the context of other evidence. | Page 7 |  |
| Implications | A4 | Consider relevance to key groups (such as policy makers, service providers and service users). Consider implications for future research. | Page 7 |  |
| Funding | | | | |
| Funding | 27 | Describe sources of funding and other support (such as supply of IPD), and the role in the systematic review of those providing such support. | Page 7 |  |

# **eTable 2. Trial-level characteristics of the seven trials included in the CaReMATCH study that included patients with angina pectoris.**

|  | | | | **ExCR** | | | | | | | **Control** |
| --- | --- | --- | --- | --- | --- | --- | --- | --- | --- | --- | --- |
| **First author (year)**  ***Acronym*** | **Main study location** | **N Total** | **N Angina pectoris (N ExCR)** | **Primary exercise mode(s)** | **Resistance training** | **Reported components** | **Setting** | **Duration (weeks)** | **Frequency (sessions per week)** | **Intensity** | **Usual care definition** |
| Campo (2020) *HULK (1-3)* | ITA | 235 | 37 (16) | Walking and calisthenics | No | Exercise, health education | Hybrid | 26 | ≥3-4 | RPE 11-13 | To underline the importance of aerobic physical activity, participants had a 15-minute visit with a study doctor and received a detailed brochure on the benefits of physical activity. |
| Hautala (2017)  *EFEX-CARE (4, 5)* | FIN | 204 | 13 (6) | Walking, running, cycling or cross-country skiing | 1-2x/week | Exercise, dietary counselling, check-up by physical therapist | Hybrid | Total: 52  Home: 52  Center: 26 | 4-5 | RPE 12-15 (aerobic), RPE 13 (resistance) | Participants randomized to usual care did not receive any individually-tailored exercise prescriptions. |
| Houle (2012) (6) | CAN | 65 | 34 (17) | Walking | No | Pedometer-based exercise, education and socio-cognitive intervention | Home | 52 | 7 | 30 min walking at moderate intensity (~100 steps/min) or RPE 11-14 | Participants received recommendations regarding physical activity, diet and medication, and had no restrictions to participate in a structured ExCR program or related healthcare professional. Participants received a blinded pedometer to assess exercise behavior every three months. |
| Lear (2014) (7-9) | CAN | 78 | 30 (16) | Participants were able to choose their preferred mode of exercise | No | Exercise, education on health, exercise and diet | Home | 16 | 3-5 | 50-80% of heart rate reserve | Participants received care from their primary care physician, and were provided internet-based resources and simple guidelines on safe exercising and healthy diet. |
| Maddison (2015)  *HEART (10-12)* | NZL | 171 | 31 (17) | Walking^a^ | No | Exercise, SMS texts and pedometer to improve exercise adherence | Home | 24 | ≥5 | RPE 11-13 (early stages of program),  RPE 13-15 (later stages of program) | All participants were free to participate in any other ExCR service or support that they wished to use, as well as encouragement to by physically active. |
| Santaularia (2017) (13-15) | ESP | 85 | 17 (6) | Cycle ergometry | 3x/week for 10 weeks | Exercise only | Centre | 10 | 3 | 75-90% of peak HR, RPE 11-15 (aerobic)  RPE 11-14 (resistance) | Participants received information on CVD risk factors. Participants were provided guidance on how to return to physical activity, and were provided instruction on breathing exercises and exercises to regain mobility, maintain muscle tone and peripheral circulation. |
| Snoek (2020)  *EU-CaRe (16, 17)* | NL | 179 | 124 (65) | Walking^a^ | No | Exercise only | Home | 26 | 5 | RPE 12-13 | Participants in the control group did not receive any form of cardiac rehabilitation but received locally-defined standard of care. |

*^a^ Walking was the primary exercise mode, but participants were allowed to deviate from walking and chose their preferred mode of exercise (e.g. cycling, swimming). ExCR exercise-based cardiac rehabilitation, HR heart rate, RPE rating of perceived exertion*

# **eTable 3. Baseline characteristics of patients with angina pectoris randomized to either ExCR or non-ExCR controls, Snoek et al. excluded.**

|  | ExCR (n = 78) | Controls (n = 84) |
| --- | --- | --- |
| Age, years (SE) | 64.61 (11.12) | 64.60 (12.13) |
| Sex, n women (%) | 16 ( 20.5) | 16 ( 19.0) |
| BMI, kg/m^2^ (SE) | 28.36 (0.52) | 28.66 (0.54) |
| LVEF, % (SE) | 53.56 (17.0) | 56.87 (15.4) |
| Current smoker, n (%) | 6 ( 7.7) | 9 ( 10.7) |
| Medication use, n (%)  *Betablockers*  *ACE-inhibitors*  *ARB*  *Antilipids*  *Diuretics*  *Antithrombotics* | 54 ( 69.2)  42 ( 53.8)  14 ( 17.9)  72 ( 92.3)  26 ( 33.3)  74 ( 94.9) | 54 ( 69.2)  42 ( 53.8)  14 ( 17.9)  72 ( 92.3)  26 ( 33.3)  74 ( 94.9) |
| Hypertension, n (%) | 45 ( 72.6) | 46 ( 65.7) |
| Dyslipidaemia, n (%) | 46 ( 74.2) | 51 ( 72.9) |
| Diabetes, n (%) | 18 ( 23.4) | 14 ( 16.7) |
| Revascularisation  *PCI, n (%)*  *CABG, n (%)* | 31 ( 56.4)  21 ( 38.2) | 29 ( 49.2)  19 ( 32.2) |

*Data were presented as means (mean difference (MD)) and standard errors (SE), medians (interquartile range (IQR)) or frequencies (%) as appropriate.*

*ACE-inhibitors angiotensin-converting enzyme inhibitors, ARB angiotensin receptor blocker, BMI body mass index, ExCR exercise based cardiac rehabilitation, LVEF left ventricular ejection fraction, PCI percutaneous coronary intervention, CABG coronary artery bypass graft.*

# **References**

1. Campo G, Tonet E, Chiaranda G, Sella G, Maietti E, Bugani G, et al. Exercise intervention improves quality of life in older adults after myocardial infarction: randomised clinical trial. Heart. 2020;106(21):1658-64.

2. Campo G, Tonet E, Chiaranda G, Sella G, Maietti E, Mazzoni G, et al. Exercise Intervention to Improve Functional Capacity in Older Adults After Acute Coronary Syndrome. J Am Coll Cardiol. 2019;74(23):2948-50.

3. Tonet E, Maietti E, Chiaranda G, Vitali F, Serenelli M, Bugani G, et al. Physical activity intervention for elderly patients with reduced physical performance after acute coronary syndrome (HULK study): rationale and design of a randomized clinical trial. BMC Cardiovasc Disord. 2018;18(1):98.

4. Hautala AJ, Kiviniemi AM, Mäkikallio T, Koistinen P, Ryynänen OP, Martikainen JA, et al. Economic evaluation of exercise-based cardiac rehabilitation in patients with a recent acute coronary syndrome. Scand J Med Sci Sports. 2017;27(11):1395-403.

5. Hautala AJ, Shavazipour B, Afsar B, Tulppo MP, Miettinen K. Machine learning models for assessing risk factors affecting health care costs: 12-month exercise-based cardiac rehabilitation. Front Public Health. 2024;12:1378349.

6. Houle J, Doyon O, Vadeboncoeur N, Turbide G, Diaz A, Poirier P. Effectiveness of a pedometer-based program using a socio-cognitive intervention on physical activity and quality of life in a setting of cardiac rehabilitation. Can J Cardiol. 2012;28(1):27-32.

7. Lear SA, Singer J, Banner-Lukaris D, Horvat D, Park JE, Bates J, et al. Randomized trial of a virtual cardiac rehabilitation program delivered at a distance via the Internet. Circ Cardiovasc Qual Outcomes. 2014;7(6):952-9.

8. Lear SA, Singer J, Banner-Lukaris D, Horvat D, Park JE, Bates J, et al. Improving access to cardiac rehabilitation using the internet: a randomized trial. Stud Health Technol Inform. 2015;209:58-66.

9. Mendell J, Bates J, Banner-Lukaris D, Horvat D, Kang B, Singer J, et al. What Do Patients Talk About? A Qualitative Analysis of Online Chat Sessions with Health Care Specialists During a "Virtual" Cardiac Rehabilitation Program. Telemed J E Health. 2019;25(1):71-8.

10. Maddison R, Pfaeffli L, Whittaker R, Stewart R, Kerr A, Jiang Y, et al. A mobile phone intervention increases physical activity in people with cardiovascular disease: Results from the HEART randomized controlled trial. Eur J Prev Cardiol. 2015;22(6):701-9.

11. Maddison R, Whittaker R, Stewart R, Kerr A, Jiang Y, Kira G, et al. HEART: heart exercise and remote technologies: a randomized controlled trial study protocol. BMC Cardiovasc Disord. 2011;11:26.

12. Maddison R, Pfaeffli L, Stewart R, Kerr A, Jiang Y, Rawstorn J, et al. The HEART Mobile Phone Trial: The Partial Mediating Effects of Self-Efficacy on Physical Activity among Cardiac Patients. Front Public Health. 2014;2:56.

13. Santaularia N, Arnau A, Jaarsma T, Torà N, Vázquez-Oliva G. Efficacy of a supervised exercise training program on five-year readmission rates in patients with acute coronary syndrome. A randomised controlled trial. Rehabilitacion (Madr). 2023;57(1):100720.

14. Santaularia N, Caminal J, Arnau A, Perramon M, Montesinos J, Abenoza Guardiola M, et al. The efficacy of a supervised exercise training programme on readmission rates in patients with myocardial ischemia: results from a randomised controlled trial. Eur J Cardiovasc Nurs. 2017;16(3):201-12.

15. Santaularia N, Caminal J, Arnau A, Perramon M, Montesinos J, Trapé J, et al. Randomized clinical trial to evaluate the effect of a supervised exercise training program on readmissions in patients with myocardial ischemia: a study protocol. BMC Cardiovasc Disord. 2013;13:32.

16. Snoek JA, Prescott EI, van der Velde AE, Eijsvogels TMH, Mikkelsen N, Prins LF, et al. Effectiveness of Home-Based Mobile Guided Cardiac Rehabilitation as Alternative Strategy for Nonparticipation in Clinic-Based Cardiac Rehabilitation Among Elderly Patients in Europe: A Randomized Clinical Trial. JAMA Cardiol. 2021;6(4):463-8.

17. Prescott E, Meindersma EP, van der Velde AE, Gonzalez-Juanatey JR, Iliou MC, Ardissino D, et al. A EUropean study on effectiveness and sustainability of current Cardiac Rehabilitation programmes in the Elderly: Design of the EU-CaRE randomised controlled trial. Eur J Prev Cardiol. 2016;23(2 suppl):27-40.
